# Supplementary material for: Active pro-vaccine and anti-vaccine groups: Their group identities and attitudes toward science
Source: PLoS One. 2021 Dec 30;16(12):e0261648. doi: 10.1371/journal.pone.0261648 (PMC8717991; doi:10.1371/journal.pone.0261648)
Supplement: Attachment — Submitted filename: Review.docx [file pone.0261648.s002.docx]

This well-written article explores group identity in pro- and anti- vaccination individuals who self-identify as being active in conversations about this issue. It finds that pro-vaccination participants have stronger group identity, both groups identify as pro-science more than not, and both take a dim view of the other’s scientific basis. With some adjustments, I think this article would be a good addition to the literature.

I note that the abstract does not really pull out the most interesting findings and urge the authors to edit it to play to the article’s strengths.

The authors build from a basis of thinking about vaccinators and non-vaccinators as people who engage in (non)scientific thinking, but at line 97-99 this appears somewhat jarring as a basis for the study, even though the authors have offered some basis for why we might think this. I think the reason it doesn’t feel right here is that pro-vax supporters are assumed to be pro-science and that this is a causal factor for their support for vaccination. I will return to this topic later. For now, I think these lines can be improved by offering evidence as to why we might believe that pro-vaxxers are also passionately pro-science. There may be academic articles that cover this point, and the authors should look for some. Failing that, they could rest on the evidence that many skeptic organisations (who are first and foremost science lovers!) engage quite heavily with the vaccination issue, including through podcasts, articles etc, and cite some examples. I feel that this evidence would make the authors’ assertion that science views matter to pro-vax views more convincing, and hence make the study’s premise more convincing.

I note that I’m a qualitative researcher so I cannot comment on the article’s quant methods. It did strike me that the N is small, and I’d like the authors to defend in the article whether the study is sufficiently powered.

Because I’m not a psychologist, I prefer explanations of results that non-quant people can understand. So, for example, at lines 212, I’d like the sentence about significant differences to actually explain who thought what. Likewise at line 221, please explain what this means in concrete language about the people in your study. At line 260, please add something that demonstrates what it means (you talk about attitudes toward science but not in which direction) Eg “meaning that pro-science people had stronger group identities, whether they were pro or anti vaccine.” This is the kind of concrete language I’m asking for throughout. At line 262, again please explain in concrete terms what the first half of this sentence is saying about your population. Including a basic explanation of what your findings actually mean in concrete terms will make your article more readable and probably more likely to be cited by others outside your discipline.

At line 290 , once again we revisit the idea that pro science motivates pro- vax behaviour / attitude / action. I’d like the authors to consider, and include in their limitations, the idea that it might swing the other way. It could also be that pro-vax people get socialised into being strong supporters of science! I raise this point because this was my journey; it might well be that of other people. Consider that causation runs the other way.

Line 313, you find that there was not a factor that clearly unites vaccine rejecters. This has been discussed in other literature – see

Kahan, D. M. 2014. Vaccine Risk Perceptions and Ad Hoc Risk Communication: An Empirical Assessment. *In* CCP Risk Perception Studies: Yale Law School; Harvard University - Edmond J. Safra Center for Ethics.

And then see critiques that he, too, might not have looked for the right thing:

Attwell, K., and Smith, D. T. 2017. Parenting as Politics: Social Identity Theory and Vaccine Hesitant Communities. International Journal of Health Governance 22 (3):183-198.

For your reflections on how the groups view each other’s scientific knowledge at lines 326-332, some other studies may be useful:

Attwell, K., Smith, D. T., and Ward, P. R. 2018. ‘The Unhealthy Other’: How vaccine rejecting parents construct the vaccinating mainstream. Vaccine 36 (12):1621-1626. doi: https://doi.org/10.1016/j.vaccine.2018.01.076.

Rozbroj, T., Lyons, A., and Lucke, J. 2019. The mad leading the blind: Perceptions of the vaccine-refusal movement among Australians who support vaccination. Vaccine 37 (40):5986-5993. doi: https://doi.org/10.1016/j.vaccine.2019.08.023.

And for your discussion of the motivations of anti-vaccinators lines 338-340, these may be useful:

Ward, P. R., Attwell, K., Meyer, S. B., Rokkas, P. R., and Leask, J. 2017. Understanding the perceived logic of care by vaccine-hesitant and vaccine-refusing parents: A qualitative study in Australia. PLoS One 12 (10).

Reich, J. 2014. Neoliberal Mothering and Vaccine Refusal: Imagined Gated Communities and the Privilege of Choice. Gender & Society 28 (5):679-704. doi: 10.1177/0891243214532711.

All suggestions are made for the authors’ benefits with no expectations that they cite any of them unless they consider them useful for their framing and wider analysis.
