# Supplementary material for: Active pro-vaccine and anti-vaccine groups: Their group identities and attitudes toward science
Source: PLoS One. 2021 Dec 30;16(12):e0261648. doi: 10.1371/journal.pone.0261648 (PMC8717991; doi:10.1371/journal.pone.0261648)
Supplement: Attachment — Submitted filename: response to reviewers.docx [file pone.0261648.s003.docx]

Dear Editor and Reviewers,

Thank you for the insightful reviews. Below are our responses to each comment.

**Editor’s comments:**

R1 and R2 are concerned that the rationale is under-developed and does nor motivate the hypotheses.

- We extensively modified the introductory part of the manuscript, including a separate part with hypotheses and their justification.

R2 also points out that the abstract needs to better represent the study

- We re-wrote the abstract to better indicate what the research is about.

Language and style needs some work

- We changed the style in many parts of the manuscript and hope that the language is better now; extensive changes requested by the Reviewers forced us to rewrite parts of the manuscript from scratch and we hope these parts are also more readable.

Please elaborate on Ariadna Nationwide Research Panel, e.g. where, which country, public daya or not?

- We provided a detailed explanation in the Method section

Provide full list of items as supplement if you have not done so already.

- We added a reference to Roccas’ social identity scale in the text. Since this is not our own scale, we don’t feel it would be ethical to reproduce it in full. All other questions are presented in full within the Methods section.

Detail the software you used

- We added information about using SPSS for analyses into the Results section

Figures appeared to be directly copied and pasted from SPSS output?

- We changed the figures to ones produced outside SPSS.

More importantly, consider running additional analyses to determine which aspect of group membership is more influential on attittudues toward science and vaccination

- We ran regression analyses to determine which aspects of group identity are most important for attitudes toward science, with interesting results.

Please elaborate on the limitations of your research. This is important.

- We added a paragraph about study limitations to the Discussion.

**Reviewer 1’s comments:**

I found the overall argumentation and structure of the article rather difficult to follow. Although several aims and hypotheses were described, I did not find a clear research question in the manuscript. Additionally, there were some inconsistencies between hypotheses, measures, and the reported findings; most notably around the concept of social identity. I will elaborate on these concerns below.

- We extensively rewrote the introduction and discussion and hope that the paper is now easier to follow.

Regarding the use of social identity in the current manuscript, some things did not become clear to me. My main concern is that the manuscript does not provide a clear reason why and how group identity/social identity should play a role among vaccine supporters and opponents. I am sure there are plenty of good reasons to study group processes here, but without a clear reason how it needs to be studied, I found it difficult to assess whether the methods were appropriate to do so. Could, for example, being personally pro- or anti vaccines not explain the same patterns in support for science ?

- We added more justification for investigating the relation between group identity and vaccine-related attitudes. The question whether being ‘personally pro/anti vaccine explain the same patterns’ is quite interesting and we cannot answer it based on our study; however we did use the well-established social identity theory, along with the social identity scale, to investigate social identity of pro-vaccine and anti-vaccine individuals, so there is very little discrepancy between theory and method. The main goal was to use this established theory to investigate the particular group of pro-vaccine and anti-vaccine people.

The methods to assess social identification match the useful distinction between the four aspects of group identity (p.4). However, two things are unclear or inconsistent here. First, the hypotheses mention “level of group identity” as a singular measure, whereas the correlations are presented for each of the four aspects of group identity. Moreover, the single reliability coefficient in the measures section suggests that indeed all 16 items are considered as a single group identity scale. When considered separately, I think a factor analysis would add to the understanding of these four sub-scales.

- We changed the wording to ‘levels’. Also, we added alpha coefficients for all subscales.

Second, the methods section lacks sample items for the identity scale. Based on the data set, I could not fully assess what the group identity exactly pertains clearly to in the items. I think this is especially important given my earlier point that it is unclear what the group identity adds in explanatory power over and above personally being pro- or anti vaccines.

- We refrained from providing a full set of items for Roccas’ scale, since it is an existing scale by another author and we don’t have the right to fully reproduce it. We did add a few sample items into the Methods section.

In the theory, I also would have liked to a see a specific discussion on this: what does a pro- or anti vaccine identity look like exactly?

🡪We have developed, as far as possible on the basis of our research, the characteristics of the group identity of both groups

I think the sub-sample drawn from the larger representative sample is fitting to test the hypotheses. The selection criteria are well-argued in the methods section. Perhaps the introduction could have been clearer that the current study concerns active/involved opponents and supporters of vaccines.

- We added a more clear statement that this is the case

Based on vaccination rates, vaccine supporters can either be an overwhelming majority group or form a minority and this can influence identification processes. Some more background information on this would help argue for the current hypotheses: how do the opponents and supporters compare to the general representative sample that they were drawn from? And what can be said beforehand about pro- and anti-vaccine groups in the current context, both practically and theoretically?

- We included these statistics in the description of our sample. Indeed, pro-vaccine individuals were thankfully a vast majority (nearly 75%), and anti-vaccine individuals were only 5% of the sample. The final sample size was constricted by the numer of anti-vaccine participants who were also active, which was about a fourth of the total anti-vaccine sample. While this may suggest that anti-vaccine individuals feel a stronger association with their minority group, these two groups operate mostly online in information bubbles, so anti-vaccine individuals may as well feel as if their group is larger than it actually is. This is why we did not state any theoretical assumptions regarding their identity based on just one group being larger or smaller than the other.

The methods and results sections contained some other puzzling information. I found the types of involvement very useful in order to get a better understanding of who the participants in the study were. However, the type of involvement was not mentioned in the introduction, hypotheses, nor methods and therefore came as a surprise to me. In the results and discussion, involvement seems to be discussed as a predictor. Additionally, the “outgroup identity” in Table 2 was not clear to me. I did not find this measure in the methods section.

🡪Thank you for this comment. Commitment was an important criterion for selecting proponents and opponents of vaccination for the study. We have now devoted more to this factor in the introduction, method and hypotheses. As for ‘outgroup identity’, we included this measure by mistake, it has now been removed from the manuscript (It was an attempt at measuring how the two groups perceive each other’s identities, but it does not fit into the theoretical framework of this paper).

For the general methodological approach of the manuscript, I think the language in the abstract, introduction, and hypotheses is not always consistent. For example, the abstract mentions “mechanisms”, suggesting group identity to potentially play a mediating role that is not test. On page 2, “understanding the predictors of […] attitudes towards vaccination” is mentioned, suggesting at least some kind of inference about predictors rather than correlations.

- We thoroughly rewrote the manuscript to avoid this type of language, as we meant ‘mechanisms’ in a theoretical context and not as a mediator; we also conducted regression analyses which allow us to speak of ‘predictors’ now, but this assumption still feels too strong.

The correlational analyses are correct as far as I can tell, but I think in some cases a regression analysis would be helpful to examine the unique contribution of, for example, the different aspects of identity in certain relations.

- We conducted regression analyses and added them to the manuscript, as their results are indeed very interesting.

Finally, I would suggest to consider addressing some conceptual and theoretical concerns in the introduction and discussion. In the introduction, I would suggest to address in more depth what the expectations are for the identification processes of opponents of vaccines. Based on the current overview of the literature, it seems that it is already clear that anti-science is not central to their ingroup or beliefs. This would make answering the question, like it is done in the current study, superfluous. Another thing I think would be important for the discussion, in what “belief in science” or “trust in science” would mean for either group. This might be hard to answer based on the measures in the study, but I could imagine that each group has “their own science” to trust, meaning that the absolute level of trust in science is less relevant than which scientific findings are believed by either group.

- We added more commentary on this in the Discussion, however it does not seem possible to clearly answer these issues with our data.

The methods section mentions that the questionnaire consists of three parts, but only two are explained

- We added a clear indication that ‘attitudes toward science’ and ‘perceived scientific knowledge’ were the third part.

I think there is a slight mismatch in the number of participants between abstract and methods section.

- Thank you for noticing this, we amended the mistake

The second hypothesis is not formally tested: it states that the correlation between level of identity and acceptance of science is stronger in the pro-vaccine group. The findings are presented in Table 4, but no conclusion is drawn about the difference between the pro- and anti-vaccine groups.

- We modified the hypothesis to reflect what we initially meant more precisely.

a phrase like “another question that arises” (p.5) needs some substantiation: where does this arise from exactly?

- We changed this wording so it does not create confusion.

**Reviewer 2’s comments:**

I note that the abstract does not really pull out the most interesting findings and urge the authors to edit it to play to the article’s strengths.

- We thoroughly rewrote the abstract to make it reflect the study better

I think the reason it doesn’t feel right here is that pro-vax supporters are assumed to be pro-science and that this is a causal factor for their support for vaccination.

- Thank you for noticing this; the relation between pro-science and vaccination support can go either way, we changed the wording to reflect it.

It did strike me that the N is small, and I’d like the authors to defend in the article whether the study is sufficiently powered.

- The small N is a result of the anti-vaccine group being, thankfully, only a small percentage of the population (in our research it was only 5% of the primary sample, we added these statistics to the text) and an even smaller percentage are active anti-vaccine individuals (about 25% of the entire anti-vaccine group). The base sample for the study was over 10500 participants, and the low final Ns show how few active anti-vaccine people there actually are.

Because I’m not a psychologist, I prefer explanations of results that non-quant people can understand.

- While we are used to presenting results in standard APA style, we tried our best to explain results in concrete terms wherever possible. However, we felt that it is necessary to keep the standard wording alongside, not to draw critique from ‘quant people’.

we revisit the idea that pro science motivates pro- vax behaviour / attitude / action. I’d like the authors to consider, and include in their limitations, the idea that it might swing the other way. It could also be that pro-vax people get socialised into being strong supporters of science!

- Of course this argument is very valid; the tendency to assume that pro-science causes vaccine support seems to stem from existing research on ‘anti-scientific beliefs’, however we agree that especially for vaccine rejection and support the relation may go the other way – people are known to reject vaccines and at the same time support science, even though this science becomes very distorted with time. We changed our wording to less causal.

Line 313, you find that there was not a factor that clearly unites vaccine rejecters. This has been discussed in other literature

- Thank you for the valuable literature suggestions. We used some of the cited papers, mainly to support the idea that vaccine rejection may have various causes.

**Reviewer 3’s comments:**

The researchers seem to base their approach on assumption that science is “the” fundamental force behind the position toward vaccines and activism around it. Thus, they measured attitude towards science and science-based social identity, and used them in their analysis. Although the authors believe that “any domain (value, symbol, attitude) can become the basis for group identity, provided that it is accepted and considered valid by the members of the group” (P.4, Line 91) they seemed to be over focused on science alone as base for group membership, and ignoring other possible sources, which might explain some possible flaws in the analysis and conclusions.

- It seems that we may have written some sections of the article in a way that caused a misunderstanding. We did not assume that pro-science or anti-science is the only cause for vaccine support or rejection. Especially we did not assume that attitudes toward science are the basis of group identity. Our main assumption was in fact that being pro-vaccine or anti-vaccine causes one to perceive themselves as part of a larger group, and identifying themselves with it. Group identity of pro-vaccine and anti-vaccine group members was our main focus of research. Whether these groups exhibit pro-scientific or anti-scientific beliefs was more of a side question which could explain people being pro-vaccine/anti-vaccine, as well as identifying themselves with each group in different ways. We changed the order of most of the paper to better indicate it, and also indicated some other possible sources of anti-vaccine beliefs.

It is true that the paper makes an excellent argument that anti-vaccine groups are not necessarily have a common negative attitude toward science , and show in the result that such attitude seems not to be the base for their group identity; However, I’m concerned about framing such results as denying the role of social identity, in general. So, if the authors aims to explore specifically the role of science-based social identity, then I would suggest making that clear, wherever needed, to avoid misleading conclusions such as “social identity of vaccination rejecters is weaker than that of vaccination supporters

- We aimed to explore the group identity (or group identification) of pro-vaccine and anti-vaccine people. It could be said that we wanted to specifically explore vaccine-based identity, attitudes toward science being one of its particular aspects (to test the hypothesis that vaccine support and vaccine rejection are caused by science support and science rejection, respectively, which turned out to be untrue. Thank you for pointing out that our paper can be interpreted in this way; we changed the wording of statements which we feel may have suggested that science is the only or main basis for group identity of vaccine supporters and rejecters.
