# Supplementary material for: Active pro-vaccine and anti-vaccine groups: Their group identities and attitudes toward science
Source: PLoS One. 2021 Dec 30;16(12):e0261648. doi: 10.1371/journal.pone.0261648 (PMC8717991; doi:10.1371/journal.pone.0261648)
Supplement: Attachment — Submitted filename: Answer to reviewers.docx [file pone.0261648.s004.docx]

Reviewer #1: In the revised manuscript “Active pro-vaccine and anti-vaccine groups: their group identities and attitudes toward science”, most of the suggestions made by reviewers seem to be addressed in one way of another. Additionally, the relevance of the topic of attitudes towards vaccination remains an important one. Nonetheless, I do think that the structure of the manuscript still contains omissions between the theoretical rationale, hypotheses, and reported findings.

One of the problems that is still in the manuscript, is how “**level of involvement**” is treated. First, the findings on level of involvement are reported quite extensively in section 3.2 of the results. Also, on line 198, it says that there is an aim of investigating this variable in relation to group identity. However, there is no specific theory, hypothesis, nor a clear measure for it. Moreover, because the participants are selected on this variable, I think section 3.2 can logically only be used as some kind of description of either group. The conclusion on page 19 (in the discussion) seems to indicate that this was the intended way of using level involvement as well.
Thank you for pointing out that our description of of „level of involvement” may be unclear. The basis for referring to this construct is the contemporary approach to group formation, in which involvement is treated as a fundamental process (Moreland and Levine, 1994). In addition to the question of defining oneself in terms of belonging to a group, contemporary analyses place a strongly emphasis on different forms of involvement in group functioning (Hogg, 1992). Therefore, in our study, the level of involvement in various activities was the criterion for selecting people into the study groups (in addition to strong pro- and anti-vaccine beliefs). Whereas in the paragraph concerning the aims of the study (on lines 189-190) we stated that the level of involvement was one of the study variables, declaring that the aim of our study was, among other things, to test its relationship with group identity and attitudes towards science. In fact, it was not a variable, but a selection criterion so the word ‘relationship’ was misleading. In the current manuscript, we have removed this phrase and also referenced the above view on the importance of involvement in group functioning.

Another problematic part of the manuscript remains the role of social identity and how it is operationalized. The reasons why social identity is used is not entirely clear even in the revised manuscript, especially when it comes to the four dimensions of social identity. In the expectations in the theoretical framework, but also in the hypothesis itself, there is no mention of difference between any of the four types of identification. Yet, these are measured and reported separately in the methods and results section. I find the regression analysis of the various social identity measures interesting but also would like to know how strongly they are related.

We have tried to better clarify the reasons for using social identity according to Roccas, and we have added more information in the manuscript about the four dimensions of social identity and the possible differences and relationships between them. Based on the results of our study, we performed additional analyses on the relationship between these dimensions of social identity (correlations between identity modes and expanded CFA).

Additionally, ideally there would be some kind of control variable for religiosity in these analyses in order to rule out alternative explanations for attitudes towards science. I do not know whether such additional measures are available.

Unfortunately, religiosity of the participants in our study was not measured. While we understand that measuring religiosity would add a possible additional level of explanation, so would measuring other variables known to relate to vaccine rejection and/or anti-science, which however would require a much larger research project, whereas we focused on group identity from the beginning.

Additionally, I think there is still work to be done in order to completely make the case for what the identities are that are measured; what their content could be. For example, some of the individual differences that are mentioned on page 3 include orthodox religiousness and moral purity concerns, both of which could easily be part of shared ingroup norms and not individual differences. The authors do not make clear how, then, the intergroup relations perspective adds to this line of research. One solution for this, I suggest, would be to provide context as to who individuals in either group could be, given the current sociopolitical situation in Poland. On page 6, there is some information on why the anti-vaccine group might have more diverse motivations and therefore identify less as anti-vaccine supporters.

As stated above, we have added more information about the four modes of group identity. Thank you for pointing out that the individual variables mentioned can be linked to norms, which are an important part of the structure of a group.

We understand that the study of group processes covers an extremely wide range of phenomena. One can focus, among other, on the individual cognitive and motivational processes leading to group behavior, on the interconnection of individual, interpersonal and social processes, on the cohesiveness of the group and its identity, etc. (Moreland & Levine, 1994).

In the manuscript, we mentioned traditional research on seeing which individual variables in a population might be predictors of attitudes toward science, toward vaccination, or toward climate change (e.g., whether climate change denial is associated with political conservatism). In contrast, we purposively recruited two groups for the study that had to meet not only the criterion of acceptance or rejection of vaccinations but also the criterion of engagement in activity related to these attitudes - to describe and compare an important group process such as group identity. In short – while existing research investigates individual differences which may be associated with group norms or group behavior (which is an assumption worth researching by itself), we investigated how much and in what manner the active pro- and anti-vaccine individuals identify with the groups they belong to – a key factor to understand whether their attitudes and beliefs are individual in nature or whether they truly identify with their respective groups

The proposition to include the current sociopolitical situation in Poland is interesting, as polls indicate that being anti-vaccine is common in supporters of the ruling Law and Justice party (a conservative, populist party), and that there is a general distrust in the government typical for the Eastern Bloc, which fuels vaccine hesitancy. However, we refrained from discussing this in our manuscript, as we did not directly measure political attitudes, so it would be pure speculation. In truth, we are not aware of any research directly describing the ‘active’ vaccine supporters and rejecters in Poland, and these groups may be different from the general anti-vaccine and pro-vaccine demographics.

In many contexts, this might seem counter-intuitive because the majority group in most situations would identify less strongly. I urge the authors to expand on this point of view by using the national context and existing literature. The Rutjens et al article on line 168 might be useful here, but then some reflection on how the current study adds to these findings is important.

We confirm the above remark. The minority group usually has a stronger group identity than a majority group; especially with regard to uncontrollable characteristics (e.g. skin color, nationality). But this does not apply to the anti-vaccine group, which is probably because people rarely think of themselves in terms of pro-vaccinationist vs. anti-vaccinationist. If it happens to them (e.g. in the course of internet discussions), pro-vaccinationists see the homogeneity of their group (everyone believes in science) which strengthens the sense of group identity, while anti-vaccinationists see the heterogeneity of their group (some of them believe in science, others do not; some are afraid of epidemics, others do not; some believe in conspiracy, others do not). We added this reasoning to the discussion of our research findings.

I would recommend changing the structure of the theoretical framework to some extent, in order to be able to see the main hypotheses and their argumentation sooner. For example, I find the hypothesis about perception of anti-science attitudes of the other group interesting, but there is barely any theorizing on it. The few lines around line 212 mention “basic rules of social cognition” while only when discussing the hypotheses, the theoretical foundations of outgroup bias are mentioned (line 231) but not discussed in depth.

We changed the structure of the paper and added more theoretical background.

All in all, quite a few confusing aspects of the initial manuscript are resolved in this revision and the correlates of pro- and anti-vaccine individuals are reported clearer now. Nonetheless, my points above still make it difficult sometimes to assess the necessity and purpose of some of the measures.

Minor issues
Space missing on line 69 and line 128
The reference on opinion-based groups should maybe already been mentioned on line 136.
The discussion paragraph starting at line 459 invokes all kinds of motivations for group membership, but I suggest making clearer what is known from previous literature versus what the findings of the current research are.
The discussion does not engage much with the theory: the findings are explained well, but it is not clear what it means for existing theory.

We spell-checked the entire manuscript, and hope to have solved all problems including missing spaces. As regards the discussion, unfortunately there is very little theory and the research is very exploratory in nature; to our knowledge, there has been no research on the identity of pro- and anti-vaccine groups, let alone strictly active ones. We added clearer statements that most of the discussion refers directly to our research. We also added some suggestions on how to merge our research with existing theory, and especially how the results of our research differ from what would be expected based on earlier research on group identity, for instance that the minority may not have a stronger identity than the majority.
